# The importance of the cerebroplacental ratio for the prognosis of neonatal outcome in AGA fetuses

**DOI:** 10.1007/s00404-022-06596-z

**Published:** 2022-05-22

**Authors:** L. Mecke, A. Ignatov, A. Redlich

**Affiliations:** University Clinic for Gynaecology, Obstetrics and Reproductive Medicine, Otto von Guericke University, Magdeburg, Germany

**Keywords:** Cerebroplacental ratio, CPR, Doppler ultrasound, AGA, Neonatal outcome

## Abstract

**Purpose:**

As a Doppler sonographic parameter, the cerebroplacental ratio (CPR) provides information about fetal hemodynamics and the redistribution of fetal blood volume in response to a metabolic change. The present study was undertaken to determine the extent to which CPR can be used as a valid parameter in routine obstetric assessment. We investigated whether CPR can be used to assess the neonatal outcome in appropriate for gestational age (AGA) fetuses and its association with secondary cesarean section due to fetal distress.

**Methods:**

In this retrospective analysis 1739 pregnant women were admitted to the University Women‘s Clinic Magdeburg, Germany, between January 2016 and December 2017. Of them, 710 AGA fetuses were eligible for analysis. SGA fetuses with an estimated fetal weight < 10th percentile were excluded from the study. The AGA fetuses were divided in two groups based on the CPR: 669 fetuses showed a normal CPR ≥ 1.08; 41 fetuses showed a decreased CPR < 1.08.

**Results:**

In our study cohort decreased CPR in AGA fetuses was associated with threefold increased rate of cesarean sections due to fetal distress (*p* < 0.001). Our data suggested that low CPR is a reliable predictor of an impaired neonatal outcome in AGA fetuses in terms of a lower birth weight, transfer to neonatology, longer length of hospitalization, and the presence of severe morbidity.

**Conclusion:**

Decreased CPR in AGA fetuses correlated with impaired neonatal outcome and secondary cesarean section due to fetal distress. The potential role of CPR for obstetric screening should be investigated in further studies.

## Introduction

Doppler sonography has been established as a safe method for assessing the fetal blood supply during pregnancy. Indications for a Doppler sonographic examination are mainly in high-risk pregnancies. As a Doppler sonographic parameter, the cerebroplacental ratio (CPR) provides information about fetal hemodynamics as well as intrauterine hypoxic states [1]. With the help of the CPR, statements can be made about a redistribution of the fetal blood volume in response to a metabolic change. The CPR is defined by the quotient of the Doppler indices of the fetal middle cerebral artery (MCA) and the umbilical artery (UA). The Pulsatility Index (PI) is usually used for this purpose [2]. If the fetus is in a hypoxic state or growth retardation, the cerebral vessels dilate to maintain blood flow to the brain (“brain-sparing effect”) [3]. This increased end-diastolic blood flow velocity is reflected in decreased Doppler indices (PI) of the MCA. Furthermore, the placental blood flow resistance increases and the end-diastolic blood flow velocity in the umbilical vessels decreases [2]. This results in increased Doppler indices of the UA. As a product of this change in perfusion, CPR decreases. CPR was first described by Arbeille et al. in 1987. In their study of 40 normal pregnancies, they showed that the cerebral vascular resistance is higher than the resistance within the placental vessels and that CPR is therefore > 1 throughout pregnancy [4]. Furthermore, CPR showed a higher sensitivity than either Doppler index (UA, MCA) alone. Since then, CPR has been the subject of numerous scientific studies [5–8].

The aim of this scientific study is to determine to what extent CPR can be integrated into routine obstetric examinations as a valid parameter for assessing fetal status. In appropriate for gestational age (AGA) fetuses the potential value of CPR to assess neonatal outcome and accurately predict a secondary cesarean section due to fetal distress was investigated. For this purpose, retrospectively collected data from the intensive pregnancy consultation (IPC) of the University Women’s Clinic Magdeburg, Germany, were analyzed.

## Methods

**Fig. 1:**
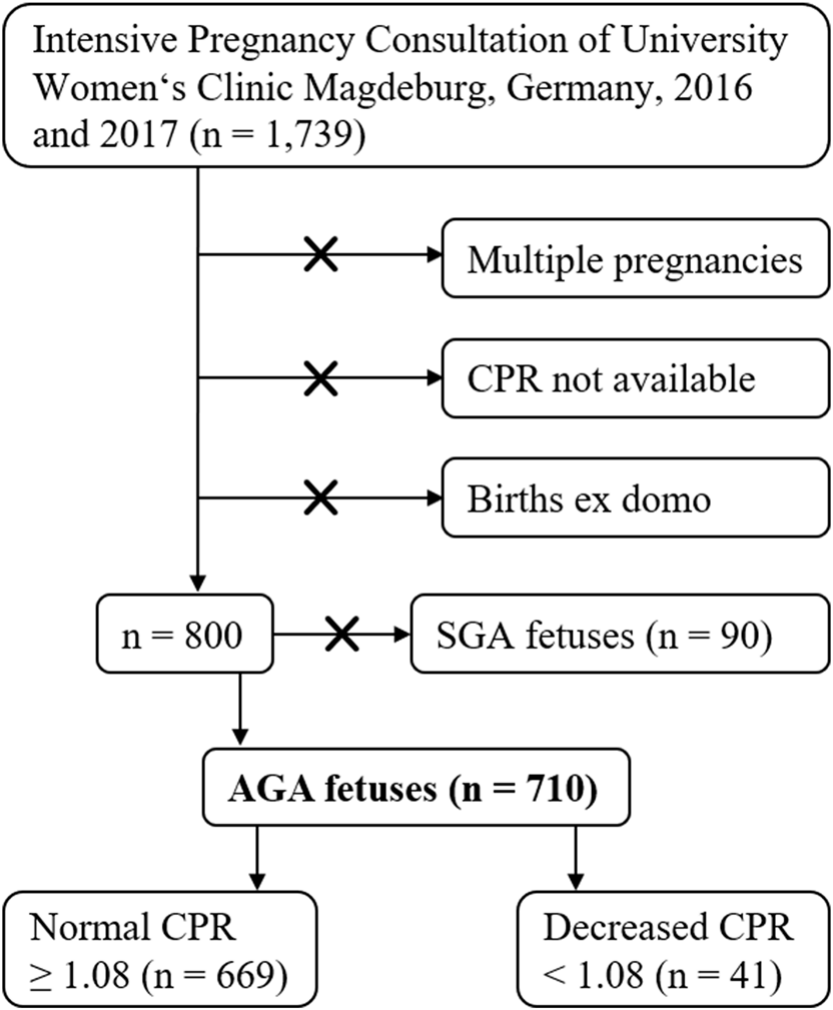
Flowchart of the data collection process. *CPR* cerebroplacental ratio, *AGA* appropriate for gestational age, *SGA* small for gestational age

The present work is a retrospective, single-center observational study. It has been approved by the Ethics Committee of the Medical Faculty of Otto von Guericke University Magdeburg. Data from all patients who had taken part in an examination as part of the intensive pregnancy consultation (IPC) at the University Women’s Clinic of Otto von Guericke University Magdeburg in 2016 and 2017 were included (*n* = 1739). Most of the patients attending this outpatient consultation decided to give birth at the University Women’s Clinic, so that outpatient and inpatient data could be correlated with each other. The aim of the data collection was to combine the data from the outpatient consultation (IPC) with the corresponding data from the birth and to evaluate them with regard to the neonatal outcome. Sonographic examinations were performed with the aid of the Voluson S8 ultrasound system (GE Healthcare) using an abdominal transducer. The examinations were performed by one and the same specialist with subspecialization in obstetrics and perinatology. Doppler sonography was used to determine the Doppler indices of the UA and MCA. CPR was then calculated. Microsoft Excel was used for data collection and processing. The most important factor for inclusion in the study was the presence of the PI of the UA as well as the PI of the MCA, as these two parameters are required for the determination of the CPR. So far, there exists no uniform cutoff value for CPR [2]. According to studies of Gramellini et al. and Odibo et al., in this study a decreased CPR < 1.08 is considered as pathological [7, 9]. This absolute cutoff value was used because of the simplified handling for the investigator. Due to this, there is no need for the use of a calculator that determines MoM values. Some patients underwent multiple examinations during the course of their pregnancy. In this case, the data of the examination with the lowest CPR in each case were included in the considerations in the sense of standardization. Multiple pregnancies were excluded from the study. Cases with births outside the University Women’s Clinic were also excluded, as there was no access to perinatal data for these. Of 1739 patients who visited the IPC in 2016 and 2017, 800 pregnancies could be included in the observations according to the inclusion and exclusion criteria mentioned above (Fig. 1). These included 710 AGA fetuses and 90 SGA fetuses. LGA fetuses (37/710; 5.2%) with an estimated fetal weight ≥ 90th percentile were grouped with AGA fetuses. The cutoff value for classification as an AGA fetus was an estimated fetal weight ≥ 10th percentile using Hadlock III formula [10, 11]. Integrated in Voluson S8 (GE Healthcare), this formula contains biparietal diameter, abdominal circumference, and femoral length. Of the 710 AGA fetuses, 669 had normal and 41 decreased prenatal CPR. AGA fetuses with normal and decreased CPR were compared in terms of neonatal outcome as well as mode of delivery (secondary cesarean section due to fetal distress). Poor neonatal outcome was associated with the following parameters: low birth weight, birth weight < 10th percentile, 1 min Apgar score < 7, 5 min Apgar score < 7, 10 min Apgar score < 7, umbilical artery pH < 7.20, umbilical artery base excess < − 8.0, transfer to neonatology, prolonged length of neonatology stay, presence of severe morbidity, or mortality. The presence of severe morbidity was defined as the presence of any of the following conditions: respiratory distress syndrome (RDS), intraventricular hemorrhage (IVH), periventricular leukomalacia (PVL), necrotizing enterocolitis (NEC), retinopathia praematurorum (ROP), bronchopulmonary dysplasia (BPD), and sepsis. Secondary cesarean section due to fetal distress refers to delivery by cesarean section due to vital risk to the fetus. Signs of this danger are a pathological CTG or Doppler examination or an abnormal micro blood test. The statistical analysis was carried out with the support of the Institute for Biometry and Medical Informatics of the Medical Faculty of Otto von Guericke University Magdeburg. First, the CPR was calculated for all cases. Subsequently, the dataset was processed and transferred from Microsoft Excel to the statistical and analysis software IBM SPSS, which was used for data analysis. For the evaluation, mainly nonparametric tests such as the Mann–Whitney *U* test and the chi-square test were used. The significance level *α* was set at 0.05 (5%). Means and standard deviations as well as percentages were rounded to the first decimal place.

## Results

In IPC, examinations were performed between the 22nd and 42nd week of pregnancy. Five examinations (5/710; 0.7%) were carried out in the second trimester while the majority of the examinations were performed in the last two months of pregnancy. The median maternal age was 30.3 years (ranged 15–46 years; Table 1).

**Table 1:** Demographic characteristics

	Normal CPR ≥ 1.08 (*n* = 669)	Decreased CPR < 1.08 (*n* = 41)	*p* value
Maternal age [years]	30.5 ± 5.3	30.4 ± 5.7	0.896
GA at examination [weeks]	**36.8 ± 2.8**	**37.6 ± 3.8**	**0.005**
Mean CPR	**1.9 ± 0.6**	**0.9 ± 0.2**	** < 0.001**
EFW percentile [%]	**45.7 ± 23.6**	**34.1 ± 22.8**	** < 0.001**
GA at delivery [weeks]	39.4 ± 1.7	38.9 ± 3.0	0.755
Birth weight [g]	**3455.7 ± 512.0**	**3044.0 ± 691.2**	** < 0.001**
Interval between examination and time of delivery [days]	**17.7 ± 15.1**	**8.9 ± 7.8**	** < 0.001**

The data are given as mean ± SD

Bold values indicate significance within the respective test procedures used. The significance level α was set at 0.05 (5 %)

*CPR* cerebroplacental ratio, *GA* gestational age, *EFW* estimated fetal weight

Of the total population, 710 cases were classified as AGA fetuses using the Hadlock III formula for fetal weight estimation. Of these, 669 fetuses had a normal CPR and 41 had a decreased CPR. These two groups were compared on the basis of the individual parameters associated with neonatal outcome. In addition, the mode of delivery was included here in terms of a secondary cesarean section due to fetal distress.

The estimated fetal weight of the AGA fetuses was on average at the 45th percentile. In the group of AGA fetuses, 5.8% (41/710) presented a decreased CPR. The mean value of the CPR values determined was 1.86. Spontaneous delivery occurred in 63.1% (448/710) of AGA fetuses. Further 15.6% (111/710) of the patients underwent a primary cesarean section, 10.1% (72/710) underwent a secondary cesarean section for fetal distress and 11.1% (79/710) got a secondary cesarean section due to other (maternal) indications.

**Fig. 2:**
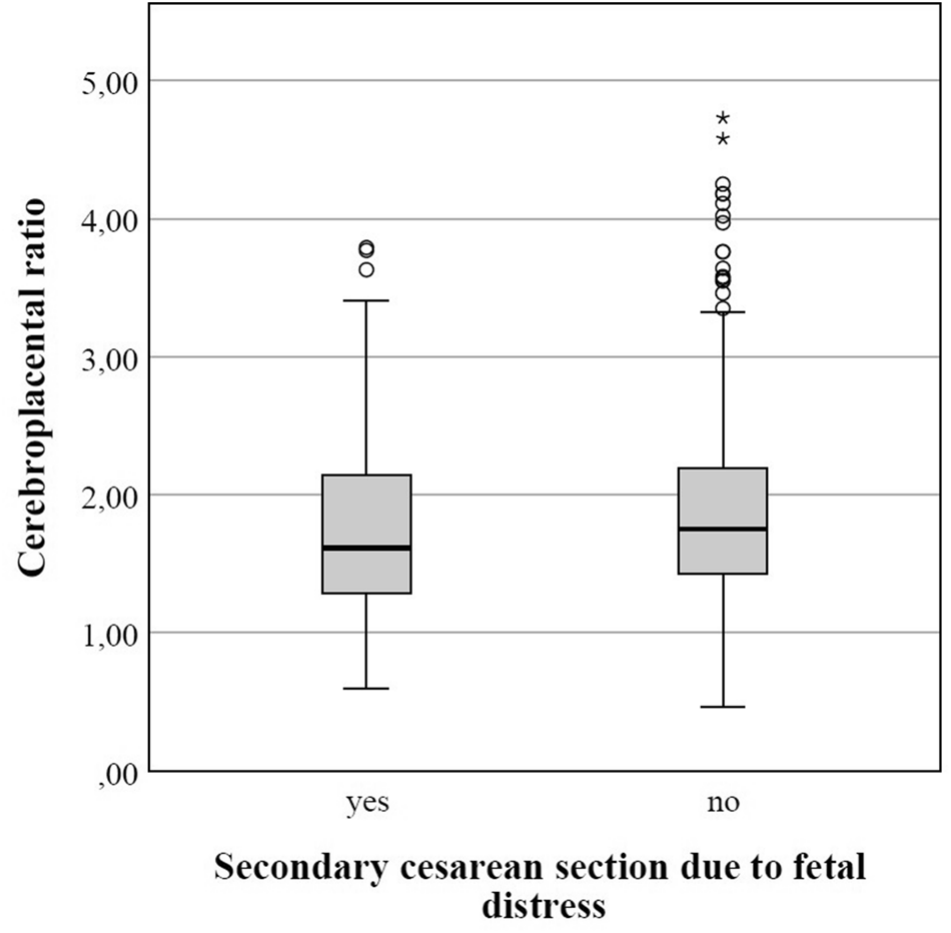
Boxplot comparing CPR of AGA fetuses with and without secondary cesarean section due to fetal distress

In the group of AGA fetuses with normal CPR, 9.0% (60/669) of cases underwent a cesarean section due to fetal distress (Table 2). Notably, in the AGA fetuses with pathological CPR, more than threefold the number (29.3%; 12/41) of cesarean sections had to be performed due to fetal distress. Therefore, abnormally low CPR in AGA fetuses was significantly associated with an increased rate of cesarean sections due to fetal distress (*p* < 0.001). Fetuses who had to be delivered by cesarean due to fetal distress showed a lower CPR prenatally (1.71 ± 0.72 vs. 1.88 ± 0.62; Fig. 2).

**Table 2:** Association of cerebroplacental ratio with secondary cesarean section due to fetal distress in 710 AGA fetuses

	Normal CPR ≥ 1.08 (*n* = 669)	Decreased CPR < 1.08 (*n* = 41)	*p* value
Secondary cesarean section for fetal distress	**60 (9.0%)**	**12 (29.3%)**	** < 0.001**^**a**^

Data are given as *n* (%)

Bold values indicate significance within the respective test procedures used. The significance level α was set at 0.05 (5 %)

*CPR* cerebroplacental ratio

Paired comparison: ^a^Fisher’s exact test

Of all cases, no newborn had a 10 min Apgar score < 7, so this parameter was omitted from the analysis. Likewise, neither intrauterine fetal death (IUFD) nor neonatal mortality occurred during the inpatient stay after birth. Furthermore, no congenital malformations were diagnosed and none of the AGA fetuses had signs of fetal growth restriction. On average, the birth weight of AGA fetuses with a normal CPR was 3456 g, while fetuses with a decreased CPR weighed an average of 3044 g after birth (*p* < 0.001, Table 3). Thus, normal-weight fetuses with reduced CPR had an average birth weight that was approximately 12% lower than fetuses with normal CPR. Fetuses with decreased CPR were over five times more likely to have an estimated fetal weight below the 10th percentile (26.8%; 11/41) than fetuses with normal CPR (*p* < 0.001). In this group, only 5.1% (34/669) of the fetuses had an estimated fetal weight below the 10th percentile. At 26.8% (11/41), fetuses with reduced CPR had to be transferred to neonatology more than twice as often as fetuses with normal CPR (11.2%; 75/669, *p* = 0.011). Also, with 7.4 days, they had an almost sevenfold longer average length of stay in neonatology than the comparison group with only 1.1 days (*p* = 0.001). Severe morbidity was diagnosed in 4.9% (2/41) of fetuses with decreased CPR. In the group of fetuses with normal CPR, there were only 0.3% (2/669) cases with severe disease (*p* = 0.018). The two groups did not differ significantly in Apgar scores, umbilical artery pH and base excess. In conclusion, decreased CPR predicted fetal impairment correctly in five neonatal outcome parameters.

**Table 3:** Association of cerebroplacental ratio with neonatal outcome in 710 AGA fetuses

		Normal CPR ≥ 1.08 (*n* = 669)	Decreased CPR < 1.08 (*n* = 41)	*p* value
Neonatal outcome parameters	Birth weight [g]	**3455.7 ± 512.0**	**3044.0 ± 691.2**	** < 0.001**^b^
	Birth weight < 10th percentile	**34 (5.1%)**	**11 (26.8%)**	** < 0.001**^a^
	1 min Apgar score < 7	14 (2.1%)	1 (2.4%)	1.000^a^
	5 min Apgar score < 7	1 (0.1%)	0 (0.0%)	1.000^a^
	Umbilical artery pH at delivery < 7.20	92 (14.2%)	4 (10.0%)	0.461^c^
	Base excess < 8.0	46 (7.1%)	1 (2.5%)	0.511^a^
	Admission to neonatology	**75 (11.2%)**	**11 (26.8%)**	**0.011**^a^
	Length of stay in neonatology [days]	**1.1 ± 5.5**	**7.4 ± 25.5**	**0.001**^b^
	Severe morbidity*	**2 (0.3%)**	**2 (4.9%)**	**0.018**^a^

Data are given as mean ± SD or *n* (%)

Bold values indicate significance within the respective test procedures used. The significance level α was set at 0.05 (5 %)

*CPR* cerebroplacental ratio

*Severe morbidity: presence of any of the following: respiratory distress syndrome (RDS), intraventricular hemorrhage (IVH), periventricular leukomalacia (PVL), necrotizing enterocolitis (NEC), retinopathia praematurorum (ROP), bronchopulmonary dysplasia (BPD), sepsis

Paired comparisons: ^b^Mann–Whitney *U* test, ^c^Pearson‘s chi-square test, ^a^Fisher‘s exact test

Multivariate regression analysis was performed to exclude confounding factors (Table 4). Several parameters of neonatal outcome were found to be dependent not only on CPR but also on estimated fetal weight, birth weight, and gestational age at birth. Thus, these variables are potential confounders. In essence, however, they support the prediction of the parameters while being unlikely to affect the influence of CPR. Only in severe morbidity, the effect of gestational age at birth is superior to that of CPR, displacing CPR in the parallel analysis.

**Table 4:** Multivariate regression analysis in prediction of neonatal outcome from pregnancy characteristics

Pregnancy characteristics	Birth weight [g]		Birth weight percentile		Admission to neonatology		Length of stay in neonatology [days]		Severe morbidity*	
	B^a^	*p* value	B^a^	*p* value	Exp(B)^b^	*p* value	B^a^	*p* value	Exp(B)^b^	*p* value
CPR	− 181.656	** < 0.001**	− 10.465	**0.001**	2.581	**0.037**	4.255	** < 0.001**	6.342	0.451
EFW percentile	12.214	** < 0.001**	0.767	** < 0.001**	1.009	0.249	0.061	0.123	0.997	0.954
Birth weight	–	**–**	–	–	0.999	**0.027**	− 0.002	** < 0.001**	1.000	0.968
GA at delivery	190.090	** < 0.001**	1.465	** < 0.001**	0.570	** < 0.001**	− 2.521	** < 0.001**	0.416	** < 0.001**

Bold values indicate significance within the respective test procedures used. The significance level α was set at 0.05 (5 %)

*CPR* cerebroplacental ratio, *EFW* estimated fetal weight, *GA* gestational age

^a^Multiple linear regression

^b^Logistic regression

*Severe morbidity: presence of any of the following: respiratory distress syndrome (RDS), intraventricular hemorrhage (IVH), periventricular leukomalacia (PVL), necrotizing enterocolitis (NEC), retinopathia praematurorum (ROP), bronchopulmonary dysplasia (BPD), sepsis

## Discussion

### Value of CPR in AGA fetuses

In AGA fetuses, CPR was shown in the present study to be a reliable predictor of an impaired neonatal outcome in terms of a lower or below 10th percentile birth weight, transfer to neonatology, longer length of stay in neonatology and the presence of severe morbidity. Compared with the Apgar score and values of the infant blood gas analysis, these parameters seem to provide more precise information about the neonatal outcome. Furthermore, a CPR < 1.08 is significantly associated with an increased risk of cesarean section due to fetal distress.

Performing a multivariate regression analysis, estimated fetal weight, birth weight and gestational age at birth were identified as potential confounders (Table 4). It is well known that these parameters can lead to impaired neonatal outcome. Thus, they support the impact of the CPR. However, the gestational age at birth has the most significant influence on the presence of severe morbidity. This can be caused by the more frequent occurrence of the considered morbidities (RDS, IVH, PVL, NEC, ROP, BPD, sepsis) in preterm infants.

The majority of current studies and recommendations for everyday clinical practice so far refers to the recording of CPR in risk groups, e.g. IUGR fetuses [12–14]. It is therefore striking that, according to the results of the present study, CPR appears to have such great significance in AGA fetuses. In 2013, Prior et al. published a prospective study in which Doppler sonographic examinations were performed on 400 AGA pregnancies immediately before birth. Of the fetuses delivered by cesarean section due to fetal compromise, 36.4% had a CPR < 10th percentile antenatally compared with only 9.5% who had a CPR ≥ 10th percentile (*p* < 0.001) [15]. No fetus with a CPR > 90th percentile had a need for cesarean section due to fetal compromise. Furthermore, fetuses with pathologically low CPR showed compromise in terms of significantly higher rates of meconium-stained amniotic fluid (*p* = 0.02) and CTG abnormalities (*p* < 0.001) [15]. In terms of neonatal outcome, fetuses with normal CPR were born with significantly higher birth weight percentiles (*p* = 0.04). These data demonstrate the high significance of CPR in identifying intrapartum risk in AGA fetuses and suggest the use of this parameter for prenatal risk stratification. Contrasting results are provided by a recent study by Buca et al. [16]. This prospective study included 553 AGA fetuses in the 37th–38th pregnancy week. In pregnancies with and without perinatal morbidity, there were no significant differences between the mean values for PI of UA (*p* = 0.486) or MCA (*p* = 0.621), nor for CPR (*p* = 0.832). In pregnancies with intrapartum complications, the mean PI of MCA was significantly lower than in the control group without complications (1.47 ± 0.4 vs. 1.61 ± 0.4; *p* = 0.0039), while there were no differences in the other Doppler parameters including CPR (*p* = 0.108) [16]. Furthermore, none of the Doppler parameters studied correlated with abnormal acid–base status at birth. Similar results were previously reported in a prospective study by Akolekar et al. [17]. The significance of CPR during a routine examination in the 37th–38th week of pregnancy was investigated on a prospective basis in over 47,000 pregnancies. A CPR < 10th percentile was associated with an unfavorable perinatal outcome, perinatal hypoxia, cesarean delivery due to fetal risk and a birth weight below the 3rd percentile [17]. However, multivariate regression analysis showed that the prediction of these adverse events was only marginally enhanced by the addition of CPR to the commonly used maternal history. Furthermore, the detection rates for individual adverse perinatal events were relatively unreliable at 13–26% and a false positive rate of about 10% [17]. According to this study, CPR does not provide any additional diagnostic advantage in the cohort of AGA fetuses as well as in the group of SGA fetuses. In contrast, according to the study by Khalil et al., lower CPR is associated with the need for surgical delivery due to fetal compromise and with admission to neonatology, regardless of the size of the fetus [18]. Overall, the scientific literature contains conflicting results on the use of CPR as a diagnostic option in AGA fetuses. The data and results of the present study confirm that CPR is highly predictive of impaired neonatal outcome in the cohort of AGA fetuses, which could be used for routine screening in the low-risk population in the future.

### Limitations

Limitations of the present study exist in particular with regard to the retrospective study design. Another limitation is the relatively small number of cases included, 710 AGA fetuses. A larger study population might make the results more valid and representative. Definitions and classifications are of great importance within this work. For example, there are no uniform thresholds and specifications for CPR, estimated fetal weight, and neonatal outcome. For CPR, there are cutoff values of < 1, < 1.08, or < 5th percentile [2]. The boundary between AGA and SGA fetuses can be drawn according to an estimated fetal weight below the 3rd, 5th, or 10th percentile. Furthermore, changes in outcomes may result from combining AGA and LGA fetuses. Neonatal outcome is also not a uniformly defined term. In order to quantify the condition of the child after birth, numerous authors use measurable parameters (e.g. birth weight, length of stay in neonatology). Attempts are sometimes made to measure the overall outcome of newborns with the help of composite scores, which combine various parameters. However, there is no scientific evidence for this type of score. It is also striking that significance with regard to an unfavorable neonatal outcome is not found in the Apgar score or in parameters of the fetal acid–base status. The results cast doubt on the significance of the two parameters mentioned with regard to fetal condition diagnostics. In particular, the Apgar score has limitations with regard to subjective factors in the assessment, interobserver variability, and the dependence of the score on maternal anaesthesia, congenital malformations, gestational age, birth trauma and fetal immaturity [19]. Consequently, it is of great importance not to consider the parameters for the fetal outcome in isolation from each other, but to assess them in their overall context. The patients included in this study were examined at different times during pregnancy. CPR values were collected from the 22nd to 42nd week of gestation in the included cases. Since CPR changes in the course of pregnancy and no distinction was made here according to the time of examination, this can lead to impaired comparability. The good interrater reliability in this study should be positively emphasized, as the examinations in the context of IPC were carried out by one and the same specialist. Since sonography is often very examiner-dependent, independence from the examiner proves to be a clear advantage in this scientific observation.

## Conclusion

The data demonstrate that decreased CPR in AGA fetuses correlates with impaired neonatal outcome in terms of a lower birth weight, transfer to neonatology, longer length of stay in neonatology and the presence of severe morbidity as well as secondary cesarean section due to fetal distress. According to our findings, we suggest the assessment of CPR in AGA fetuses within the third trimester ultrasound screening and/or as a part of birth planning in 34th–36th week of gestation. Overall, the results are promising for the future integration of CPR into a reliable obstetric screening. This will require further, especially prospective, studies. Further investigations of the present study will evaluate the informative value of CPR in SGA fetuses and compare CPR with previously established screening methods such as estimated fetal weight and CTG.
